# Halogen Bonds Formed between Substituted Imidazoliums and N Bases of Varying *N*-Hybridization

**DOI:** 10.3390/molecules22101634

**Published:** 2017-09-29

**Authors:** Steve Scheiner

**Affiliations:** Department of Chemistry and Biochemistry, Utah State University, Logan, UT 84322-0300, USA; steve.scheiner@usu.edu; Tel.: + 1-435-797-7419

**Keywords:** NBO, molecular electrostatic potential, AIM, trimethylamine, imine, nitrile

## Abstract

Heterodimers are constructed containing imidazolium and its halogen-substituted derivatives as Lewis acid. N in its sp^3^, sp^2^ and sp hybridizations is taken as the electron-donating base. The halogen bond is strengthened in the Cl < Br < I order, with the H-bond generally similar in magnitude to the Br-bond. Methyl substitution on the N electron donor enhances the binding energy. Very little perturbation arises if the imidazolium is attached to a phenyl ring. The energetics are not sensitive to the hybridization of the N atom. More regular patterns appear in the individual phenomena. Charge transfer diminishes uniformly on going from amine to imine to nitrile, a pattern that is echoed by the elongation of the C-Z (Z=H, Cl, Br, I) bond in the Lewis acid. These trends are also evident in the Atoms in Molecules topography of the electron density. Molecular electrostatic potentials are not entirely consistent with energetics. Although I of the Lewis acid engages in a stronger bond than does H, it is the potential of the latter which is much more positive. The minimum on the potential of the base is most negative for the nitrile even though acetonitrile does not form the strongest bonds. Placing the systems in dichloromethane solvent reduces the binding energies but leaves intact most of the trends observed in vacuo; the same can be said of ∆G in solution.

## 1. Introduction

Although the hydrogen bond (H-bond) is the noncovalent force that has arguably received the greatest attention over the years [1,2,3,4,5,6,7], the halogen bond (XB) is not far behind and its study continues to grow apace [8,9,10,11,12,13,14,15,16]. These interactions have been studied in numerous situations [17], varying from gas phase [18,19,20], to solution and solid state [21,22,23,24,25,26,27], superfluid He droplets [28], self-assembled nanostructures [29,30,31], clathrate cages [32], and on a solid/liquid interface [33]. Other environments include molecular capsules [34], and within the confines of proteins and other biological systems [35,36,37,38]. XBs are not mere passive players, but actively participate in synthesis and catalysis [13,39,40,41], or induce the formation of complex structures such as triple helicates [42], and even maximize power conversion efficiency of solar cells [43].

H-bonds and XBs are of comparable strength, and arise from a similar set of contributing factors. As in H-bonds, electron donors/halogen acceptors can be varied, not only classical lone pairs, but π-bonds and aromatic systems [36,44,45,46,47,48], carbenes [49], σ-bonds [50], and even metal atoms [47,51,52]. Numerous studies [20,53,54] have led to a set of general rules concerning halogen bonds. There is a tendency for this bond to strengthen as one moves down the periodic table: Cl < Br < I; F is a reluctant participant. This strengthening arises from progressively greater contributions from Coulombic attraction, charge transfer, and dispersive forces. As in the H-bond, the propensity of the electron donor group, the Lewis base, to release a certain amount of density drives the halogen bond toward enhanced strength. The halogen bond tends to be a bit more sensitive to angular deformations than is the H-bond [55,56], with generally similar stretching dependence [57,58].

A number of pressing issues still require resolution. In the first place, Lewis acids that are of aromatic character have not been examined extensively. Nor has there been much consideration of halogen donors that bear a full positive charge. The imidazolium species represents a strong candidate for study of these matters, especially given its prominence [41,59,60,61,62,63,64,65,66] within the context of anion receptors and catalysis as well as in ionic liquids. How strong a halogen bond can arise when the H of the positively charged imidazolium is replaced by a halogen atom? Does the pattern of enhanced binding noted for neutral Lewis acids remain in force for these ionic species? Again in the context of ionic Lewis acids, how do the various halogen bonds compare with the H-bond of the unsubstituted imidazolium Can any of these interactions be enhanced by extra conjugation if the imidazolium species is connected with a phenyl ring? In connection with the Lewis base, how does the hybridization of the N atom on the electron donor affect the halogen bond? What is the nature of any alkylation effect associated with substitution of N? Given the competition that may be present between H-bonds and XBs [67,68,69,70,71,72], and the sensitivity of this competition to the nature of the solvent [73], it is judicious to examine these issues not only in a fundamental way in the gas phase, but also within solvent.

## 2. Systems and Methods

In addition to imidazolium ImH^+^ as Lewis acid, the H atom was replaced by each of several halogens Cl, Br, and I as electron-accepting atom. Ammonia (NH_3_) was taken as a Lewis base with sp^3^ hybridization of the electron donor N atom. The hybridizations sp^2^ and sp were sampled via an imine MeN=CHMe and acetonitrile N≡CMe, respectively. The effects of replacing the H atoms of NH_3_ by methyl were elucidated by comparison with NMe_3_. Perturbing effects arising from the attachment of each of the imidazolium species to a phenyl ring were examined as well.

Calculations were carried out via the Gaussian-09 [74] program suite at the MP2/aug-cc-pVDZ level; the aug-cc-pVDZ-PP pseudopotential from the EMSL library [75,76] was used for I so as to account for relativistic effects. A substantial body of past work has verified the accuracy of this theoretical approach [77,78,79,80,81,82,83,84,85,86,87,88,89] for closely related systems.

All geometries were fully optimized with no restriction, and verified as true minima with no imaginary vibrational frequencies. The binding energy of each heterodimer, ∆E, was defined as the difference between the energy of the complex and the sum of the energies of individually optimized monomers. The standard counterpoise [90] procedure corrected for basis set superposition error. In addition to gas phase, solvent effects were included via the polarizable conductor calculation model (CPCM) [91], taking dichloromethane as solvent. Standard physical chemistry equations [92] were applied to evaluate free energies at 298 K. Molecular electrostatic potential maps were visualized via the Chemcraft program [93] with greater quantification provided by Multiwfn [94]. The Natural Bond Orbital (NBO) technique [95] was used to provide quantitative measures of charge transfer. The topology of the electron density was assessed via the Atoms-in-Molecules (AIM) [96,97] procedure, utilizing the AIMALL [98] program. The interaction energy was dissected using symmetry-adapted perturbation theory (SAPT) methods [99,100,101].

## 3. Results

### 3.1. Geometries and Energetics

**Figure 1:**
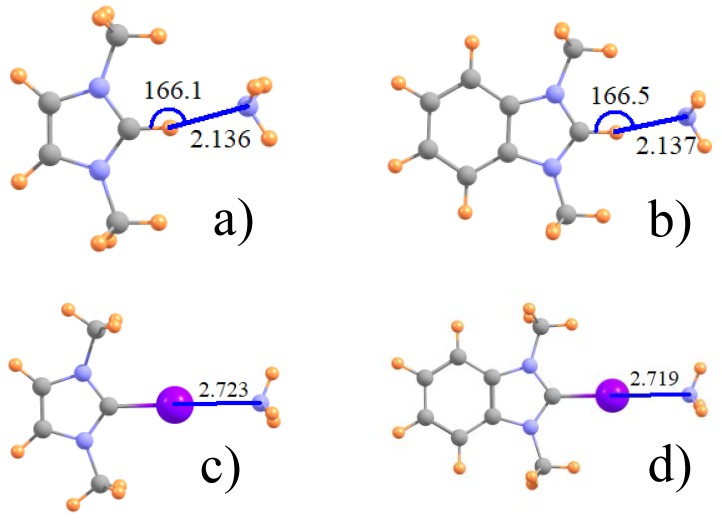
Optimized geometry of (**a**) ImH^+^ and (**b**) its phenyl derivative with NH_3_; and (**c**,**d**), their I-substituted analogues. Distances in Å and angles in degs.

The geometries of some typical complexes are displayed in Figure 1 for both the unsubstituted ImH^+^ proton donor (**1a**) and its I-substituted analog (**1c**) which engages in a halogen bond with NH_3_. The right side of Figure 1 exhibits the analogous geometries wherein a phenyl ring is attached to the Im. All halogen bonds, whether Z=I, Br, or Cl are fully linear with θ(CZ···N) = 180°, whereas the H-bonds illustrated in Figure 1a,b deviate by some 14° from full linearity. This asymmetry may be influenced by the presence of a weak CH···N H attractive interaction to one of the methyl groups, with R(N···H) ~ 3.0 Å. The intermolecular separations of the various dimers are reported in the first two rows of Table 1, where it may be noted that H-bonds are considerably shorter than the respective halogen bonds. Contrary to the growing size of the halogen atoms, the halogen bond length diminishes in the order Cl > Br > I. It may also be observed that the inclusion of the phenyl ring on Im has little discernible effect on these intermolecular distances.

Many of these same patterns are evident in the binding energies listed in Table 2. Again, addition of the phenyl ring yields little perturbation of the results. The halogen bonding energies are sensitive to the nature of the Z atom, nearly doubling as Z changes from Cl to Br to I. The H-bond is roughly comparable to the Br halogen bond, intermediate between Cl and I.

**Figure 2:**
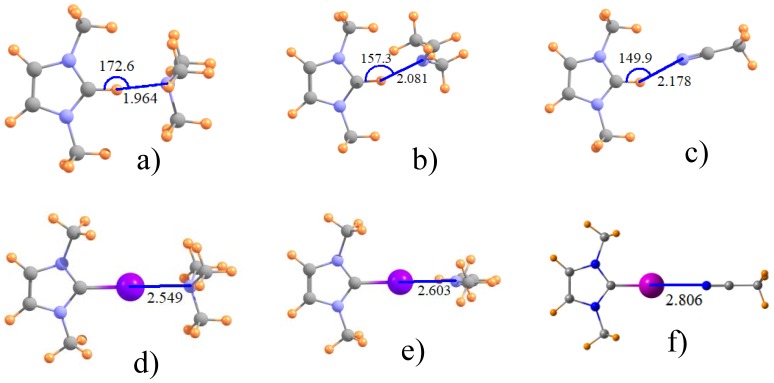
Optimized geometry of ImH^+^ with (**a**) NMe_3_; (**b**) imine MeN=CHMe and (**c**) nitrile MeCN; (**d**–**f**) illustrate I-substituted analogues.

The effect of adding alkyl groups to the amine may be discerned by inspecting the data for NMe_3_ in the third rows of Table 1 and Table 2. Some relevant geometries are displayed in Figure 2a,d, where again halogen bonds are linear and H-bonds are not. The addition of these methyl substituents to the N cause quite significant contraction of the intermolecular distances, by as much as 0.22 Å for Z=Br, but only half that for Z=Cl. There is likewise a strengthening of these bonds, from 0.7 kcal/mol for Z=Cl to 4.5 kcal for Z=I, corresponding to 8% and 30%, respectively. Again, Z=H behaves much like Z=Br in terms of quantitative changes of some 20%.

Another issue of interest concerns the hybridization of the Lewis acid N atom. The next two rows of Table 1 and Table 2 relate the data for sp^2^-hybridized imine, followed in the last row by the nitrile group with its sp hybridization, which may be compared to the sp^3^ N in the amines. The intermolecular distances for the MeN=CHMe imine is shorter by some 0.05–0.12 Å than for NH_3_, but longer when compared to the fully methylated NMe_3_ amine. The latter may perhaps be taken as a better point of comparison, given the presence of methyl groups on the imine as well. (As before, addition of a phenyl to the imidazole ring has little effect.) The change to sp hybridization in the nitrile in the last row of Table 1 yields a further increment in the distance. Like the H-bond to the ImH^+^ in Figure 1a, those involving other hybridizations of the N are similarly nonlinear, while the halogen bonds retain their linearity. Turning next to the energetics in Table 2, the transformation from NMe_3_ to MeN=CHMe and thence to N≡CMe has mixed results. While slightly strengthening the Cl-bonds, a weakening occurs for both Br and I, particularly the latter. This trend for Br and I is consistent with the lengthening halogen bond whereas the strengthening of the Cl bond is at odds with the same elongation pattern. One last point relates to the nonlinearity of the H-bonds. As is apparent in Figure 2, this nonlinearity increases as one progresses from amine to imine and thence to nitrile.

In many experimental situations, the interactions in question would occur in solution, rather than in the gas phase. Immersion of the various systems into dichloromethane solvent yielded the binding energies listed in Table 3. As is typical of this sort of interaction, the binding is weakened in solvent. The reduction in binding energy lies in the range of 5–11 kcal/mol. This decrement is on the order of roughly half, with drops varying between 40% and 80%. On the other hand, the trends in Table 3 mimic those in Table 2 to a large extent. Whether gas phase or solution, Cl < Br < I, with H roughly comparable to Br. Interactions are strengthened by the addition of methyl groups to the amine, and weakened in the order sp^3^ > sp^2^ > sp. The latter trend is more strictly enforced in solution than in the gas phase where there are a couple of violations, as noted above, most particularly for Z=Cl.

Incorporation of both solvent and thermal/entropic effects enables the estimation of the free energies of binding for the various complexes in solution. Unlike ∆E, the values of ∆G in Table 4 are all positive, indicating that formation of these dimers is not a spontaneous process at 25 °C. Although the trends are muted to some extent, one still sees the general trend that Cl bonds are the weakest and I bonds the strongest (i.e., least positive). The hybridization pattern is similar to that noted for ∆E, but there are exceptions and most of the differences from one system to the next are smaller with respect to ∆G.

### 3.2. Underlying Electronic Structure Patterns

There are a number of means of analyzing the wave functions so as to determine the fundamental reasons why the energetics behave as they do. The AIM formalism analyzes the topography of the electron density and identifies bond paths between atoms. The density of the critical point along each bond path offers a quantitative measure of the strength of each bond. This density is reported in Table 5 for the bond connecting the Z atom on the imidazolium that acts as electron acceptor and the N donor atom. For each row, there is a clear increasing trend Cl < Br < I, with the value for H roughly comparable to that for Br. This pattern is identical to that noted for the binding energies. As one scans down each column of Table 5, the highest densities occur for the trimethylamine donor and the nitrile is the weakest: NMe_3_ > MeN=CHMe > NH_3_ > N≡CMe. In other words sp^3^ > sp^2^ > sp, with the additional aspect that methyl substituents add to ρ_BCP_, when compared to H. This pattern does not match up precisely with energetics, where the trends were less clear. While Z=I fits this pattern, there is much less distinction with respect to N hybridization for the other Z atoms, as a slight preference for the sp-hybridized N is in evidence for some.

**Figure 3:**
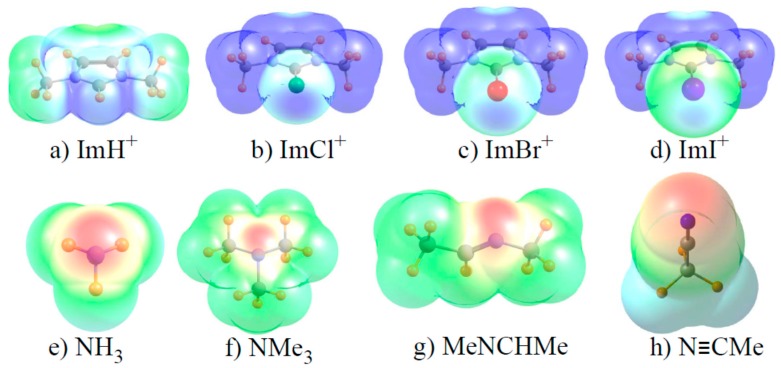
Molecular electrostatic potential surrounding each monomer on an isodensity surface of ρ = 0.001 au. Minimum (red) and maximum (blue) regions correspond respectively to (**a**) 0.15–0.20; (**b**–**d**) 0.10–0.15; (**e**–**h**) −0.08–+0.08. All potentials drawn on surface corresponding to 1.5 times the van der Waals radius of each atom.

It is recognized that halogen and related bonds contain a strong electrostatic component, particularly in cases such as these where one of the two subunits bears a full charge. Accordingly, the molecular electrostatic potential (MEP) of each monomer was evaluated, and is visualized in Figure 3. The blue regions indicate the most positive areas of each MEP, with the most negative/least positive shown in red. With respect to the imidazoliums in the top row of Figure 3, one sees a positive blue region near each Z atom, corresponding to the so-called σ-hole (although there are more intense blue regions in other segments of some of these molecules). Note, however, that the blue region appears to become less intense as Cl transitions to Br and then to I, opposite to the pattern of increasing bond strength. With regard to the N electron acceptors in the bottom row of Figure 3, the most negative red region corresponds roughly to the position of the N lone pair. This red area is largest and most intense for the nitrile in Figure 3h. In contrast to the energetic data where methyl substituents enhance the binding, the red region is more intense for NH_3_ than for NMe_3_.

A more quantitative assessment of the MEP can be derived by searching for the points of extrema on each MEP. The values of these extrema are reported in Table 6 as V_s,max_ for the imidazoliums and V_s,min_ for the Lewis bases. In contrast to the diagrams in Figure 3, and in better keeping with energetic patterns, V_s,max_ grows clearly more positive as Cl < Br < I. On the other hand this quantity is largest of all for Z=H although its bond strength is clearly less than for Z=I. The values of V_s,min_ for the bases are largest for NH_3_ and N≡CMe, and smallest for NMe_3_ and the imine. This trend runs counter to the stronger binding of the methylated amine, and would incorrectly suggest particularly strong binding by the nitrile.

In addition to electrostatic attraction, these bonds also benefit from a charge transfer component, and in particular the transfer from the lone pair of the Lewis base N into the σ*(C-Z) antibonding orbital of the acid. The energetic consequence of the latter transfer is displayed in Table 7 as E(2) as computed via the NBO algorithm. These quantities faithfully follow the Cl < Br < I paradigm, and again Z=H is roughly comparable to Z=Br. The dependence upon base is consistent with the sp^3^ > sp^2^ > sp order and reflects the strengthening effect of methylation (with the exception of Z=H). In short, the E(2) charge transfer quantity behaves very much as one might predict from simple chemical intuition, although the ultimate binding energy appears to represent a more complex combination of elements.

As one result of the aforementioned charge transfer into the σ*(C-Z) antibonding orbital, one would anticipate a weakening and thus elongation of the equilibrium length of this bond. These stretches are indeed observed, and their amounts displayed in Table 8. Like E(2) itself, there is again the growth of this stretch in the Cl < Br < I order in all cases, with Z=H situated between Cl and Br. The elongation is sensitive to the base as well: The sp^3^ > sp^2^ > sp order is consistent with E(2), dropping precipitously from amine to nitrile. The alkylation of the base adds to this effect.

In addition to its stretch, the weakening of the C-Z bond occasioned by the formation of the dimer ought to also cause a reduction in the vibrational stretching frequency ν_s_(C-Z). However, it must be understood that this normal mode is not a pure C-Z stretch but also contains elements of other atomic motions. As an example, the C-I stretching motion in ImI^+^, occurring at 1178 cm^−1^, is accompanied by a distortion of the entire Im ring. With this caveat, it is nonetheless instructive to examine the perturbations introduced into this vibrational mode by the complexation with each of the various Lewis bases. The changes listed in Table 9 are all to lower frequencies, consistent with the C-Z bond weakening. One sees enormously larger red shifts for Z=H, when compared to halogen substituents. And these shifts for ImH^+^ fit the earlier data with sp^3^ > sp^2^ > sp, and a magnifying effect arising from alkylation. This same trend also appears for the halogens, even if numerically much smaller. The much larger shifts occurring for Z=H may occur as this particular C-H stretching mode is much purer for ImH^+^, with considerably less contamination from Im ring distortions than occurs for halogen substituents.

Another means of analyzing the interactions can be achieved through a decomposition of the total interaction energy into its various components. The SAPT components of the interaction between the four ImZ^+^ cations and NH_3_ are reported in Table 10. All components grow in the order Cl < Br < I, with the exchange repulsion and induction showing the most rapid increase, and the electrostatic attraction grows a bit more slowly. These patterns are consistent with the data reported above. The slower increase of the electrostatic energy is consistent with the V_s,max_ values in Table 6, and the much more rapid growth of the induction mirrors the NBO measures of interorbital charge transfer in Table 7. Note, however, that the magnitude of ES for ImH^+^ in Table 10 is unexpectedly small when compared to its value of V_s,max_, and likewise for the smallness of the corresponding IND component for ImH^+^, when compared to NBO E(2). As a bottom line, it might be concluded that ES is the dominant attractive force for the H-bonded ImH^+^···NH_3_, there is more of a balance between ES and IND for Z=Cl and Br, but induction plays the most important role for ImI^+^···NH_3_.

## 4. Conclusions

There are a number of patterns emerging from the data presented here. The amount of charge transfer from the N lone pair to the σ*(CZ) antibonding orbital increases sharply as the halogen becomes heavier: Cl < Br < I. The replacement of the halogen by a H atom provides data roughly comparable to Br. The transition from amine to imine to nitrile, with diminishing n in sp^n^ hybridization, diminishes the charge transfer. The replacement of methyl groups on the base by H atoms reduces the latter effects. These patterns are reflected also in the elongation induced in the C-Z bond of ImZ^+^ by complexation with a Lewis base. These trends are carried over to the red shifts of the ν(C-Z) bond stretching frequencies, with the caveat that these shifts are many times larger for Z=H than for the halogenated Lewis acids. An independent means of assessing the intermolecular bond strength, the density at the bond critical point, confirms these patterns.

The electrostatic component of the interaction can be interpreted via molecular electrostatic potentials. Consistent with charge transfer trends, V_s,max_ on the isodensity surface increases with the size of the X atom, but the largest potential occurs for Z=H, rather than for I. V_s,min_ on the Lewis base is most negative for the nitrile, with little to distinguish between the amine and imine. Also in contrast to charge transfers, the replacement of methyl groups by H makes this quantity more negative, encouraging a stronger rather than weaker interaction. Pictorial representations of the MEP are consistent with the most negative potentials around NH_3_ and MeCN. On the other hand, these diagrams suggest that the intensity of the positive region in the Lewis acid diminishes with the size of the halogen, contradicting the trend in V_s,max_. Inconsistencies between MEP and the final interaction energies are not uncommon [65,87,102,103,104,105,106]. There are clearly issues at play other than simple Coulombic forces. One issue is the greater dispersion energy that will likely arise for heavier halogens, or for replacement of H by Me.

Considering certain inconsistencies from one component of the interaction to the next, it is not surprising to note certain irregularities in the H/X bond strengths of these systems, as measured by energetics. On one hand, there is consistency in that the halogen bond is enhanced in the order Cl < Br < I, and that the H-bond is generally similar to the Br-bond. There is also agreement that the bond is strengthened by methyl substituents on the Lewis base. On the other hand, most of these interactions exhibit little dependence upon the hybridization of this N atom. The trends observed in the gas phase energetics are largely preserved when the system is immersed in dichloromethane solvent, despite a reduction in the interaction energies. Inclusion of vibrational and entropic effects lead to positive values of ∆G at 298 K, but largely maintain the same trends.

It may be expected that an anion will bind more strongly to an imidazolium cation than will a neutral molecule such as those considered here. For example, recent calculations [107] estimate the binding energy of Cl^−^ to a receptor containing a pair of imidazoliums to be 165 kcal/mol in the gas phase, many times larger than the value of 14 kcal/mol for acetonitrile. The former value may be inflated by the dicationic nature of the receptor. Indeed, another work [65] considered the single interaction of ImH^+^ with Cl^−^, and found an interaction energy of 25 kcal/mol, nearly double the same quantity for acetonitrile. 

As noted earlier, the fusion of a simple phenyl ring to the imidazolium had little effect upon its binding properties. On the other hand, the addition of electron-withdrawing or releasing substituents to this phenyl ring might be anticipated to exert appropriate effects. For example, perfluorinated PhImZ^+^ would likely pull electron density away from the Z atom, improving its ability to accept electrons from the Lewis base, and thereby strengthen the interaction.

## Figures and Tables

**Table 1:** Optimized R(N···Z) intermolecular distance (Å) of substituted imidazoliums with N-bases.

Acid	Base	H	Cl	Br	I
ImZ^+^	NH_3_	2.136	2.826	2.803	2.723
PhImZ^+^	NH_3_	2.137	2.829	2.806	2.719
ImZ^+^	NMe_3_	1.964	2.712	2.583	2.549
ImZ^+^	MeN=CHMe	2.081	2.773	2.708	2.603
PhImZ^+^	MeN=CHMe	2.087	2.774	2.712	2.595
ImZ^+^	N≡CMe	2.178	2.820	2.833	2.806

**Table 2:** Binding energies (kcal/mol) of substituted imidazoliums with N-bases in vacuum.

Acid	Base	H	Cl	Br	I
ImZ^+^	NH_3_	−11.07	−8.42	−10.71	−15.13
PhImZ^+^	NH_3_	−10.87	−8.32	−10.56	−15.00
ImZ^+^	NMe_3_	−13.36	−9.13	−12.81	−19.60
ImZ^+^	MeN=CHMe	−13.36	−9.57	−12.34	−17.97
PhImZ^+^	MeN=CHMe	−13.11	−9.48	−12.21	−17.89
ImZ^+^	N≡CMe	−14.27	−10.15	−11.66	−14.69

**Table 3:** Binding energies (kcal/mol) of substituted imidazoliums with N-bases in CH_2_Cl_2_ solvent.

Acid	Base	H	Cl	Br	I
ImZ^+^	NH_3_	−4.10	−2.69	−4.58	−8.71
PhImZ^+^	NH_3_	−4.52	−2.94	−4.91	−9.33
ImZ^+^	NMe_3_	−4.94	−3.85	−6.28	−12.05
ImZ^+^	MeN=CHMe	−5.01	−3.21	−5.07	−9.38
PhImZ^+^	MeN=CHMe	−5.54	−3.42	−5.45	−10.13
ImZ^+^	N≡CMe	−3.51	−2.26	−3.28	−5.35

**Table 4:** Free energies (kcal/mol) of binding substituted imidazoliums with N-bases in CH_2_Cl_2_ solvent at 298 K.

Acid	Base	H	Cl	Br	I
ImZ^+^	NH_3_	4.83	5.34	4.21	1.10
PhImZ^+^	NH_3_	4.84	4.68	4.31	0.62
ImZ^+^	NMe_3_	6.08	5.59	4.69	0.09
ImZ^+^	MeN=CHMe	5.59	6.30	5.55	2.34
PhImZ^+^	MeN=CHMe	6.24	8.58	4.87	1.87
ImZ^+^	N≡CMe	5.30	5.16	4.38	4.63

**Table 5:** AIM bond critical point density, ρ_BCP_ (au), along Z···N noncovalent bond for ImZ^+^ Lewis acids.

Base	H	Cl	Br	I
NH_3_	0.0225	0.0180	0.0221	0.0314
NMe_3_	0.0325	0.0243	0.0383	0.0487
MeN=CHMe	0.0242	0.0199	0.0271	0.0407
N≡CMe	0.0173	0.0156	0.0179	0.0229

**Table 6:** V_s,max_ and V_s,min_ (kcal/mol) for Lewis acids and bases, respectively, on isopotential surface corresponding to ρ = 0.001 au.

Lewis Acid	V_s,max_	Lewis Base	V_s,min_
ImH	124.31	NH_3_	−38.02
ImCl	97.14	NMe_3_	−32.12
ImBr	100.35	MeN=CHMe	−32.25
ImI	110.91	N≡CMe	−38.09

**Table 7:** NBO values of E(2) (kcal/mol) for charge transfer from N lone pair of base to σ*(C-Z) antibonding orbital of ImZ^+^ Lewis acid.

Base	H	Cl	Br	I
NH_3_	15.76	4.93	9.61	20.31
NMe_3_	15.10	5.97	16.74	32.73
MeN=CHMe	12.64	4.69	11.04	26.78
N≡CMe	7.97	2.76	5.35	11.03

**Table 8:** Change in r(C-Z) bond length (Å) caused by complexation with ImZ^+^ Lewis acid.

Base	H	Cl	Br	I
NH_3_	0.015	0.006	0.016	0.048
NMe_3_	0.025	0.013	0.042	0.088
MeN=CHMe	0.014	0.008	0.024	0.068
N≡CMe	0.004	0.000	0.005	0.022

**Table 9:** Change in C-Z stretching vibration frequency (cm^−1^) in ImZ^+^ Lewis acid caused by complexation.

Base	H	Cl	Br	I
NH_3_	−180.2	−3.9	−5.5	−2.7
NMe_3_	−375.9	−11.8	−16.9	−7.9
MeN=CHMe	−169.4	−7.7	−6.7	−7.8
N≡CMe	−28.8	+0.8	−0.8	−2.2

**Table 10:** SAPT energy components (kcal/mol) of ImZ^+^···NH_3_ complexes

Component	H	Cl	Br	I
ES	−14.70	−12.53	−16.98	−28.33
EXCH	12.52	19.68	36.27	65.83
IND	−5.25	−10.92	−24.02	−60.36
DISP	−3.48	−3.33	−4.27	−6.35
total	−10.92	−7.11	−9.00	−29.21
